# Bio-heat response of skin tissue based on three-phase-lag model

**DOI:** 10.1038/s41598-020-73590-3

**Published:** 2020-10-02

**Authors:** Qiao Zhang, Yuxin Sun, Jialing Yang

**Affiliations:** Institute of Solid Mechanics, School of Aeronautic Science and Engineering, Beihang University, Beijing, 100191 People’s Republic of China

**Keywords:** Biomedical engineering, Biophysics

## Abstract

In this article, the thermal response of skin tissue is investigated based on three-phase-lag (TPL) model of heat conduction. The governing equation of bio-heat conduction is established by introducing both the TPL model of heat conduction and a modified energy conservation equation. The analytical solution is obtained by adopting the method of separation of variables and a parametric study on temperature responses in TPL model is carried out. It is shown that the TPL model can predict both the diffusion and wave characteristics of bio-heat conduction. Increasing the phase-lag of thermal displacement gradient would result in the rise of thermal propagation speed and decrease the temperature in affected zone. The perfusion rate of arterial blood has no obvious effect on thermal propagation velocity and thermal propagation lagging. Increasing of the rate of blood perfusion contributes to decreasing the temperature of steady state.

## Introduction

The heat transfer in biological tissue such as skin is a complicated process because it involves the heat conduction in solid tissues, solid–liquid convection between blood and solid tissues, metabolic heat generation and blood perfusion^1^. The deep research of bio-heat transfer mechanism in skin tissue is significant and helpful for heat therapy in medical treatment.

Generally speaking, three kinds of bio-heat transfer models for skin tissue are usually adopted in recent literatures^2^: Pennes model^3^, thermal wave (C-V) model^4,5^ and dual phase lag (DPL) model^6^. The first model of bio-heat transfer in biological tissue was presented by Pennes, which is based on the Fourier’s law,

1 $$ \vec{q}\left( {\vec{r},t} \right) = - k\nabla T\left( {\vec{r},t} \right) $$

where $\vec{q}\left( {\vec{r},t} \right)$ is heat flux vector, *T* is absolute temperature, *k* is thermal conductivity, $\vec{r}$ is the location vector and *t* represents time.

Because of the simplicity of analysis, Pennes model was widely used in bio-heat transfer. However there are some inherent disadvantages for the bio-heat conduction model as it assumes that the thermal propagation speed is infinite^7^. As a matter of fact, heat is always found to propagate with a finite speed in living biological tissues within which there are highly non-homogeneous inner structures.

Considering the concept of a finite thermal propagation speed, Cattaneo^4^ and Vernotte^5^ almost simultaneously modified the Fourier’s law to form the thermal wave model which is also called C-V heat conduction model, which is expressed as

2 $$ \vec{q}\left( {\vec{r},t} \right) + \tau \frac{{\partial \vec{q}\left( {\vec{r},t} \right)}}{\partial t} = - k\vec{\nabla }T\left( {\vec{r},t} \right) $$

The equation indicates that the heat flux and temperature gradient occur at different moments for a specified position. The delay time between the heat flux and temperature gradient is defined as the thermal relaxation time $\tau$. The thermal wave theory ensures that the heat flux is the consequence of the temperature gradient and temperature gradient is the cause so that there is a strong path dependency for the temperature gradient^4,5^.

Later, Tzou^8^ proposed a dual phase lag (DPL) model by introducing the phase lag of heat flux which is the time delay due to the fast transient effect of thermal inertia. The conduction relation between heat flux vector and temperature gradient can be expressed as

3 $$ \vec{q}\left( {\vec{r},t + \tau_{q} } \right) = - k\vec{\nabla }T\left( {\vec{r},t + \tau_{T} } \right) $$

where $\tau_{q}$ is the phase lag time for the heat flux vector, and $\tau_{T}$ is the phase lag time for the temperature gradient.

The above three heat conduction models are widely used by researchers and the effects of various thermophysical properties on thermal responses have been discussed adequately. For example, Lin and Li^9^ obtained the analytical solutions of bio-heat transfer for skin tissue with general boundary conditions in the Pennes, C-V and DPL models by using separation variable method. They discussed the effect of phase-lags, boundary conditions and heat conduction models on temperature and thermal damage. Xu et al.^10^ presented a comprehensive literature review pertinent to the bio-thermomechanical behavior of skin tissue which reviewed the fundamental concepts, methodologies and various thermophysical properties. Based on the Laplace transform method, Liu^11^ theoretically investigated the thermal behavior in a living tissue subjected to constant, sinusoidal, or step surface heating with the thermal wave model of bio-heat transfer. Babaei and Chen^12^ utilized the hyperbolic heat conduction model in a functionally radius graded hollow cylinder and analyzed the effects of thermal relaxation time and nonhomogeneous indices by numerical inversion of the Laplace transform. Akbarzadeh and Chen^13^ studied heat conduction in one-dimensional functionally graded media based on the DPL model.

Recently, Choudhuri^14^ established Three-phase-lag (TPL) constitutive model (4) by introducing the phase-lag of heat flux vector, phase-lag of temperature gradient and phase-lag of thermal displacement gradient in heat conduction law based on a model of thermoelasticity developed by Green and Naghdi^15,16^. If the thermal conductivity and phase-lags are assumed as zero, the reduced heat conduction model would be obtained like equation (5) which could be regarded as a special C-V heat conduction model when the thermal relaxation time $\tau$ approaches to infinity. Hence the three-phase-lag model based on G-N model could probably predict the non-Fourier phenomenon in medium with large phase lag time, like sand and biological skin. It is necessary to investigate the bio-heat transfer behavior of biological skin tissue induced by external heat source in TPL model.

4 $$ \vec{q}\left( {\vec{r},t + \tau_{q} } \right) = - \left[ {k\nabla T\left( {\vec{r},t + \tau_{T} } \right) + k^{*} \nabla v\left( {\vec{r},t + \tau_{v} } \right)} \right] $$

5 $$ \frac{{\partial \vec{q}}}{\partial t}\left( {\vec{r},t} \right) = - k^{*} \nabla T\left( {\vec{r},t} \right) $$

Besides the temperature gradient which serves as a constitutive variable in C-V and DPL heat conduction models, thermal displacement gradient is considered as a third constitutive variable in TPL model. The introduction of TPL model provides a general theoretical heat conduction model and a new approach to predict thermal response and damage of structures^17^. Akbarzadeh et al.^17^ studied heat conduction in a functionally graded, infinitely-long hollow cylinder based on TPL model. Quintanilla and Racke^18^ obtained conditions on the phase-lag and material parameters to guarantee the exponential stability for two cases in the heat conduction theory with three-phase-lag. And generalized thermoelasticity based on three-phase-lag effect is investigated^19–21^. Kar et al.^19^ studied the thermoelastic responses in a functionally graded orthotropic hollow sphere due to a sudden temperature change in context of three-phase-lag model of generalized thermoelasticity. Kothari et al.^20^ attempted to find the fundamental solutions in the context of generalized thermo-elasticity with three phase-lags. Mukhopadhyay et al.^21^ presented a thorough analysis of effects of phase lags on wave propagation and investigated the nature of distributions of different fields in a thick plate based on thermoelasticity with three phase-lags and thermoelasticity of type GN-III (without any phase lag). Biswas and coauthors^22–25^ published a set of researches based on TPL model, such as the propagation of Rayleigh waves in orthotropic thermos-elastic half-space, the solution in closed form for an electro-magneto thermoelastic coupled two-dimensional problem in orthotropic half space, the various heat source responses in a transversely isotropic hollow cylinder, and free vibration of homogenous isotropic cylinder panel with voids. Li et al.^26^ investigated the transient thermoelastic responses of bi-layered skin tissue with temperature-dependent thermal material parameters in the context of generalized thermoelasticity without energy dissipation.

To the authors’ knowledge, the three-phase-lag effect hasn’t been considered in bio-heat transfer model in biological skin tissue. With this motivation in mind the aim of present analysis is to investigate the bio-heat transfer model with three-phase-lag effect in biological tissue. The governing equation is established and an analytical solution is obtained by using the method of separation of variables. A complete and comprehensive analysis on the relative parameters and comparison are presented.

## Theoretical formulations

Choudhuri^14^ introduced a constitutive heat flux relation with three phase lags

6 $$ \vec{q}\left( {\vec{r},t + \tau_{q} } \right) = - \left[ {k\nabla T\left( {\vec{r},t + \tau_{T} } \right) + k^{*} \nabla v\left( {\vec{r},t + \tau_{v} } \right)} \right] $$

in which $\vec{q}$, *T* and *v* are heat flux vector, absolute temperature and thermal displacement which satisfies $\dot{v} = T$, respectively. $\tau_{q}$, $\tau_{T}$ and $\tau_{v}$ are the phase-lags of heat flux vector, temperature gradient and thermal displacement gradient, respectively. *t* is time and $\vec{r}$ is the position coordinate vector. *k* and k^*^ are the thermal conductivity and the rate of thermal conductivity, respectively. $\nabla$ denotes the gradient operator. Equation (6) can be rewritten into the following form like DPL model.

7 $$ \vec{q}\left( {\vec{r},t + \tau_{q} } \right) = - k\nabla \left[ {T\left( {\vec{r},t + \tau_{T} } \right) + \frac{{k^{*} }}{k}v\left( {\vec{r},t + \tau_{v} } \right)} \right] $$

in which the term ($T + k^{*} v/k$) can be viewed as an new integral variable and therefore Eq. (7) could be regarded as a kind of extension of DPL heat conduction equation (3).

Now Taylor’s series expansion of Eq. (7) up to the first-order terms in relative with $\tau_{q}$, $\tau_{T}$ and $\tau_{v}$ leads to the following generalized heat conduction equation at a point $\vec{r}$ and time *t*.

8 $$ \left( {1 + \tau_{q} \frac{\partial }{\partial t}} \right)\vec{q} = - k\vec{\nabla }\left[ {\left( {1 + \tau_{T} \frac{\partial }{\partial t}} \right)T + \left( {1 + \tau_{v} \frac{\partial }{\partial t}} \right)\left( {\frac{{k^{*} }}{k}v} \right)} \right] $$

By using the relation $\dot{v} = T$, equation (8) can be rewritten into the following form which is comprised of two variables, namely the temperature *T* and heat flux $\vec{q}$.

9 $$ \left( {\frac{\partial }{\partial t} + \tau_{q} \frac{{\partial^{2} }}{{\partial t^{2} }}} \right)\vec{q} = - \left[ {k\tau_{T} \frac{{\partial^{2} }}{{\partial t^{2} }} + \left( {k + k^{*} \tau_{v} } \right)\frac{\partial }{\partial t} + k^{*} } \right]\nabla T $$

For k^*^ = 0, Eq. (9) would reduce to the following equation

10 $$ \left( {\frac{\partial }{\partial t} + \tau_{q} \frac{{\partial^{2} }}{{\partial t^{2} }}} \right)\vec{q} = - k\left( {\frac{\partial }{\partial t} + \tau_{T} \frac{{\partial^{2} }}{{\partial t^{2} }}} \right)\nabla T $$

which can be simplified into the DPL heat conduction equation by integrating with time.

11 $$ \left( {1 + \tau_{q} \frac{\partial }{\partial t}} \right)\vec{q} = - k\left( {1 + \tau_{T} \frac{\partial }{\partial t}} \right)\nabla T $$

Furtherly, it could reduce to the thermal wave model of Eq. (2) when the phase-lag of temperature gradient is set to zero, namely $\tau_{T} = 0$. And in the case of k^*^ = 0 and $\tau_{q} = \tau_{T} = 0$, equation (9) would be reduced to the heat conduction equation of Fourier’s law.

According to Pennes model^3^, the energy conservation equation of classical bio-heat transfer could be written as

12 $$ \rho_{t} c_{t} \frac{\partial T}{{\partial t}} = - \nabla \cdot \vec{q} + \omega_{b} \rho_{b} c_{b} \left( {T_{a} - T} \right) + Q_{m} + Q_{laser} $$

where the subscript ‘*t*’ denotes skin tissue and ‘*b*’ represents arterial blood flowing into tissue. *ρ* and *c* are density and specific heat, respectively. *ω*_*b*_ is the perfusion rate of blood which represents the volume of blood flowing into unit volume skin tissue per unit time. *T*_*a*_ is the reference temperature of arterial blood entering skin tissue, which keeps the same as constant temperature of inner body, hence the second term on the right-hand side is regarded as heat exchange between arterial blood and the local tissue. *Q*_*m*_ and *Q*_*laser*_ are heat generation contributions to tissue which are provided by metabolic process and external laser heating, respectively.

It is worthwhile to mention that Eq. (12) cannot be used directly for the TPL model of bio-heat transfer, and a modification is needed to introduce. The necessity and reason of the modification and substitution to energy conservation equation is illustrated in Appendix. According to Eq. (7), the term $\left( {T + k^{*} v/k} \right)$ is viewed as an integral variable, so it takes place of the temperature *T* in Eq. (12) as well. Then the following energy equation would be adopted in the present study,

13 $$ \rho_{t} c_{t} \frac{\partial }{\partial t}\left( {T + \frac{{k^{*} }}{k}v} \right) = - \nabla \cdot \vec{q} + \omega_{b} \rho_{b} c_{b} \left[ {\left( {T_{a} - T} \right) + \frac{{k^{*} }}{k}\left( {v_{a} - v} \right)} \right] + Q_{m} + Q_{laser} $$

It is easy to find that Eq. (13) could reduce to the classical energy conservation Eq. (12) in the case of k^*^ = 0. The thermal displacement *v* can be eliminated from Eq. (13) by using the relation $\dot{v} = T$ and a partial differential equation of $T$ and $\vec{q}$ can be obtained as

14 $$ \rho_{t} c_{t} \left( {\frac{\partial }{\partial t} + \frac{{k^{*} }}{k}} \right)\frac{\partial T}{{\partial t}} + \omega_{b} \rho_{b} c_{b} \left( {\frac{\partial }{\partial t} + \frac{{k^{*} }}{k}} \right)\left( {T - T_{a} } \right) = - \nabla \cdot \frac{{\partial \vec{q}}}{\partial t} + \frac{\partial }{\partial t}\left( {Q_{m} + Q_{laser} } \right) $$

By eliminating heat flux $\vec{q}$ from Eqs. (9) and (14), the bio-heat transfer equation for temperature based on TPL model in homogeneous skin tissue can be obtained as

15 $$ \begin{gathered} \hfill \rho_{t} c_{t} \left( {1 + \tau_{q} \frac{\partial }{\partial t}} \right)\left( {\frac{{\partial^{2} T}}{{\partial t^{2} }} + \frac{{k^{*} }}{k}\frac{\partial T}{{\partial t}}} \right) + \omega_{b} \rho_{b} c_{b} \left( {1 + \tau_{q} \frac{\partial }{\partial t}} \right)\left( {\frac{\partial }{\partial t} + \frac{{k^{*} }}{k}} \right)\left( {T - T_{a} } \right) = \\ \hfill \left[ {k\tau_{T} \frac{{\partial^{2} }}{{\partial t^{2} }} + \left( {k + k^{*} \tau_{v} } \right)\frac{\partial }{\partial t} + k^{*} } \right]\nabla^{2} T + \left( {\frac{\partial }{\partial t} + \tau_{q} \frac{{\partial^{2} }}{{\partial t^{2} }}} \right)\left( {Q_{m} + Q_{laser} } \right) \\ \end{gathered} $$

When k^*^ = 0, equation (15) can also reduce to DPL bio-heat conduction by integral over time, which is

16 $$ \begin{aligned} & \rho_{t} c_{t} \frac{\partial T}{{\partial t}}\left( {1 + \tau_{q} \frac{\partial }{\partial t}} \right) + \omega_{b} \rho_{b} c_{b} \left( {1 + \tau_{q} \frac{\partial }{\partial t}} \right)\left( {T - T_{a} } \right) = k\left( {1 + \tau_{T} \frac{\partial }{\partial t}} \right)\nabla^{2} T \\ & \quad + \left( {1 + \tau_{q} \frac{\partial }{\partial t}} \right)\left( {Q_{m} + Q_{laser} } \right) \\ \end{aligned} $$

By introducing the temperature increment *θ* = *T – T*_*a*_ and considering *T*_*a*_ keeps constant, equation (15) can be changed into the following equation solved in the paper.

17 $$ \begin{aligned} & \tau_{q} \rho_{t} c_{t} \frac{{\partial^{3} \theta }}{{\partial t^{3} }} + \left[ {\rho_{t} c_{t} \left( {1 + \tau_{q} \frac{{k^{*} }}{k}} \right) + \tau_{q} \omega_{b} \rho_{b} c_{b} } \right]\frac{{\partial^{2} \theta }}{{\partial t^{2} }} + \left[ {\rho_{t} c_{t} \frac{{k^{*} }}{k} + \omega_{b} \rho_{b} c_{b} \left( {1 + \tau_{q} \frac{{k^{*} }}{k}} \right)} \right]\frac{\partial \theta }{{\partial t}} \\ & \quad + \omega_{b} \rho_{b} c_{b} \frac{{k^{*} }}{k}\theta = \left[ {k\tau_{T} \frac{{\partial^{2} }}{{\partial t^{2} }} + \left( {k + k^{*} \tau_{v} } \right)\frac{\partial }{\partial t} + k^{*} } \right]\nabla^{2} \theta + \left( {\frac{\partial }{\partial t} + \tau_{q} \frac{{\partial^{2} }}{{\partial t^{2} }}} \right)\left( {Q_{m} + Q_{laser} } \right) \\ \end{aligned} $$

## Solution to the problem

In this section, the method of separation of variables is taken to solve the problem and obtain analytical solution for the three-phase-lag bio-heat transfer model. Two kinds of boundary conditions are under consideration to stimulate two load cases in reality.

### Case I: Constant temperature on the surface of skin

This case stimulates the bio-heat transfer of skin tissue when the external surface of skin is abruptly attached to a high temperature object and the temperature of outer surface is viewed as the temperature of the object’s temperature. A step heating on the outer surface is adopted, which means the outer surface is heated to reach the specific temperature of *T*_0_ and then it keeps constant temperature for simplicity. The inner surface contacts with the arterial blood of the body, so it is assumed to keep the same constant temperature as the body temperature *T*_*a*_. The thickness of the skin is *L*. The boundary conditions are defined as

18 $$  \left\{ {\begin{array}{*{20}l}    {\theta \left( {0,t} \right) = \theta _{0}  \cdot {\text{H}}\left( t \right)} \hfill  \\    {\theta (L,t) = 0} \hfill  \\   \end{array} } \right.  $$

where *H*(*t*) is the Heaviside function and *θ*_0_ = *T*_0_—*T*_*a*_.

### Case II: Constant heat flux exerted on the surface of skin

This case predicts the bio-heat transfer behavior when the highly absorbing skin suffers from a laser light irradiance. In such a case, the absorbed laser energy could be viewed as boundary heat flux ^27,28^ as

19 $$ q\left( {0,t} \right) = \frac{{\partial \theta \left( {0,t} \right)}}{\partial x} = \phi_{in} \left( {1 - R_{d} } \right) $$

where *R*_*d*_ is the diffuse reflectance of light at the irradiated surface. *φ*_*in*_ is the intensity of laser irradiance varying with time as follows.

20 $$ \phi_{in} = Q_{0} \cdot {\text{H}}\left( t \right) $$

where *Q*_0_ is the maximum intensity of laser. The internal surface is still assumed as constant temperature *T*_*a*_ like the second term of Eq. (18). Therefore the boundary condition for the case of constant heat flux could be written as

21 $$  \left\{ {\begin{array}{*{20}l}    {\frac{{\partial \theta \left( {0,t} \right)}}{{\partial x}} = \phi _{{in}} \left( {1 - R_{d} } \right)} \hfill  \\    {\theta (L,t) = 0} \hfill  \\   \end{array} } \right. $$

### Solution of the problem

In practice, a one-dimensional model of the tissue is sufficient as the size of laser beam is much larger than the thickness of tissue. The *x*-axis points inwards the skin tissue with the original point positioned on the outer surface of tissue. The temperature increment governing equation (17) turns to the following form considered in this paper.

22 $$ \begin{aligned} & \tau_{q} \rho_{t} c_{t} \frac{{\partial^{3} \theta }}{{\partial t^{3} }} + \left[ {\rho_{t} c_{t} \left( {1 + \tau_{q} \frac{{k^{*} }}{k}} \right) + \tau_{q} \omega_{b} \rho_{b} c_{b} } \right]\frac{{\partial^{2} \theta }}{{\partial t^{2} }} + \left[ {\rho_{t} c_{t} \frac{{k^{*} }}{k} + \omega_{b} \rho_{b} c_{b} \left( {1 + \tau_{q} \frac{{k^{*} }}{k}} \right)} \right]\frac{\partial \theta }{{\partial t}} \\ & \quad + \omega_{b} \rho_{b} c_{b} \frac{{k^{*} }}{k}\theta = \left[ {k\tau_{T} \frac{{\partial^{2} }}{{\partial t^{2} }} + \left( {k + k^{*} \tau_{v} } \right)\frac{\partial }{\partial t} + k^{*} } \right]\frac{{\partial^{2} \theta }}{\partial x} + \left( {\frac{\partial }{\partial t} + \tau_{q} \frac{{\partial^{2} }}{{\partial t^{2} }}} \right)\left( {Q_{m} + Q_{laser} } \right) \\ \end{aligned} $$

The initial conditions are set as

23 $$ \left. {\theta (x,t)} \right|_{t = 0} = 0,\;\;\;\;\;\;\left. {\frac{\partial \theta }{{\partial t}}(x,t)} \right|_{t = 0} = 0,\;\;\;\;\;\;\left. {\frac{{\partial^{2} \theta }}{{\partial t^{2} }}(x,t)} \right|_{t = 0} = 0 $$

To deal with the nonhomogeneous boundary condition and find out the steady state of eigen mode of temperature increment, the following equation is deduced by setting all the time derivative terms in Eq. (22) to be zero,

24 $$ \omega_{b} \rho_{b} c_{b} \frac{{k^{*} }}{k}\theta = k^{*} \nabla^{2} \theta $$

The general solution of above equation is $\theta = c_{1} e^{\lambda x} + c_{2} e^{ - \lambda x}$.

Firstly with the boundary condition (18) of case I, the solution of Eq. (24) can be expressed as

25 $$ \varphi \left( x \right) = \left( {\frac{{e^{ - \lambda x} - e^{{\lambda \left( {x - 2L} \right)}} }}{{1 - e^{ - 2\lambda L} }}} \right)\theta_{0} $$

where $\lambda = \sqrt {\omega_{b} \rho_{b} c_{b} /k}$. The steady state solution that satisfies boundary conditions equation (18) can be expressed as

26 $$ \theta_{2} \left( {x,t} \right) = \varphi \left( x \right) \cdot {\text{H}}\left( t \right) $$

Therefore temperature increment can be expressed as the following form.

27 $$ \theta \left( {x,t} \right) = \theta_{1} \left( {x,t} \right) + \varphi \left( x \right) \cdot {\text{H}}\left( t \right) $$

By substituting Eq. (27) into Eqs. (22), (18) and (23), the following equation for the transient temperature increment $\theta_{1} \left( {x,t} \right)$ is obtained.

28 $$ \begin{aligned} & \tau_{q} \rho_{t} c_{t} \frac{{\partial^{3} \theta_{1} }}{{\partial t^{3} }} + \left[ {\rho_{t} c_{t} k_{q} + \tau_{q} \omega_{b} \rho_{b} c_{b} } \right]\frac{{\partial^{2} \theta_{1} }}{{\partial t^{2} }} + \left[ {\rho_{t} c_{t} \frac{{k^{*} }}{k} + \omega_{b} \rho_{b} c_{b} k_{q} } \right]\frac{{\partial \theta_{1} }}{\partial t} + \omega_{b} \rho_{b} c_{b} \frac{{k^{*} }}{k}\theta_{1} \\ & \quad = \left[ {k\tau_{T} \frac{{\partial^{2} }}{{\partial t^{2} }} + \left( {k + k^{*} \tau_{v} } \right)\frac{\partial }{\partial t} + k^{*} } \right]\nabla^{2} \theta_{1} + \left( {\frac{\partial }{\partial t} + \tau_{q} \frac{{\partial^{2} }}{{\partial t^{2} }}} \right)\left( {Q_{m} + Q_{laser} } \right) \\ & \quad \quad - \varphi \left( x \right)\left\{ \begin{gathered} \tau_{q} \rho_{t} c_{t} \frac{{\partial^{3} {\text{H}}\left( t \right)}}{{\partial t^{3} }} + \left[ {\rho_{t} c_{t} k_{q} + \omega_{b} \rho_{b} c_{b} \left( {\tau_{q} - \tau_{T} } \right)} \right]\frac{{\partial^{2} {\text{H}}\left( t \right)}}{{\partial t^{2} }} \hfill \\ + \left[ {\rho_{t} c_{t} + \omega_{b} \rho_{b} c_{b} \left( {\tau_{q} - \tau_{v} } \right)} \right]\frac{{k^{*} }}{k}\frac{{\partial {\text{H}}\left( t \right)}}{\partial t} \hfill \\ \end{gathered} \right\} \\ \end{aligned} $$

where $k_{q} = 1 + \tau_{q} {{k^{*} } \mathord{\left/ {\vphantom {{k^{*} } k}} \right. \kern-\nulldelimiterspace} k}$. And the corresponding boundary conditions and initial conditions of $\theta_{1} \left( {x,t} \right)$ are

29 $$ {\mathbf{B}}{\mathbf{.C}}{\mathbf{.}}\;\;\;\left\{ \begin{gathered} \theta_{1} (0,t) = 0 \hfill \\ \theta_{1} (L,t) = 0 \hfill \\ \end{gathered} \right. $$

30 $$ {\mathbf{I}}{\mathbf{.C}}{\mathbf{.}}\;\;\;\left\{ \begin{gathered} \left. {\theta_{1} (x,t)} \right|_{t = 0} = - \varphi \left( x \right) \cdot {\text{H}}\left( 0 \right) \hfill \\ \hfill \\ \left. {\frac{{\partial \theta_{1} }}{\partial t}(x,t)} \right|_{t = 0} = - \varphi \left( x \right) \cdot {\dot{\text{H}}}\left( 0 \right) \hfill \\ \left. {\frac{{\partial^{2} \theta_{1} }}{{\partial t^{2} }}(x,t)} \right|_{t = 0} = - \varphi \left( x \right) \cdot {\ddot{\text{H}}}\left( 0 \right) \hfill \\ \end{gathered} \right. $$

The characteristic equation of Eq. (28) is

31 $$ \tau_{q} \rho_{t} c_{t} \frac{{\partial^{3} \theta_{1} }}{{\partial t^{3} }} = k^{*} \frac{{\partial^{2} \theta_{1} }}{{\partial x^{2} }} $$

By utilizing the method of separation of variables, the solution of Eq. (31) is expressed as

32 $$ \theta_{1} \left( {x,t} \right) = Y\left( x \right)B\left( t \right) $$

Substituting Eq. (32) into Eq. (31) and considering the boundary conditions equation (29), one can obtain the eigen-function as

33 $$ Y_{n} \left( x \right) = \sin \left( {\lambda_{n} x} \right) $$

where $\lambda_{n} = {{n\pi } \mathord{\left/ {\vphantom {{n\pi } L}} \right. \kern-\nulldelimiterspace} L},\;\;n = 1,2,3 \ldots$ is the eigen-value. And the eigen function satisfies

34 $$ \int_{0}^{L} {Y_{n} \left( x \right)Y_{m} \left( x \right){\text{d}}x} = \left\{ \begin{gathered} 0\begin{array}{*{20}c} {} & {n \ne m} \\ \end{array} \hfill \\ \frac{L}{2}\begin{array}{*{20}c} {} & {n = m} \\ \end{array} \hfill \\ \end{gathered} \right. $$

Therefore the temperature increment $\theta_{1} \left( {x,t} \right)$ could be expressed as

35 $$ \theta_{1} \left( {x,t} \right) = \sum\limits_{n = 1}^{\infty } {Y_{n} \left( x \right)B_{n} \left( t \right)} $$

Applying Eq. (35) into Eq. (28), the time terms *B*_*n*_(*t*) needs to satisfy the following equation

36 $$ a\frac{{\partial^{3} B_{n} \left( t \right)}}{{\partial t^{3} }} + b\frac{{\partial^{2} B_{n} \left( t \right)}}{{\partial t^{2} }} + c\frac{{\partial B_{n} \left( t \right)}}{\partial t} + dB_{n} \left( t \right) = f\left( t \right) $$

where the coefficients are defined as

37 $$ \begin{aligned} a & = \tau_{q} \rho_{t} c_{t} \hfill \\ b & = \rho_{t} c_{t} k_{q} + \tau_{q} \omega_{b} \rho_{b} c_{b} + k\tau_{T} \lambda_{n}^{2} \hfill \\ c & = \rho_{t} c_{t} \frac{{k^{*} }}{k} + \omega_{b} \rho_{b} c_{b} k_{q} + \left( {k + k^{*} \tau_{v} } \right)\lambda_{n}^{2} \hfill \\ d & = \omega_{b} \rho_{b} c_{b} \frac{{k^{*} }}{k} + k^{*} \lambda_{n}^{2} \hfill \\ \end{aligned} $$

And the nonhomogeneous term is expressed as

38 $$ \begin{aligned} f\left( t \right) & = \left( {\frac{\partial }{\partial t} + \tau_{q} \frac{{\partial^{2} }}{{\partial t^{2} }}} \right)\frac{2}{L}\int_{0}^{L} {Y_{n} \left( x \right)} \left( {Q_{m} + Q_{laser} } \right){\text{d}}x \\ &\quad- \frac{2}{L}\int_{0}^{L} {Y_{n} \left( x \right)} \varphi \left( x \right){\text{d}}x\left\{ \begin{gathered} \tau_{q} \rho_{t} c_{t} \frac{{\partial^{3} {\text{H}}\left( t \right)}}{{\partial t^{3} }} + \left[ {\rho_{t} c_{t} k_{q} + \omega_{b} \rho_{b} c_{b} \left( {\tau_{q} - \tau_{T} } \right)} \right]\frac{{\partial^{2} {\text{H}}\left( t \right)}}{{\partial t^{2} }} \hfill \\ + \left[ {\rho_{t} c_{t} + \omega_{b} \rho_{b} c_{b} \left( {\tau_{q} - \tau_{v} } \right)} \right]\frac{{k^{*} }}{k}\frac{{\partial {\text{H}}\left( t \right)}}{\partial t} \hfill \\ \end{gathered} \right\} \\ \end{aligned} $$

The corresponding initial conditions are obtained by substituting Eq. (35) into Eq. (30), which is

39 $$ \left\{ \begin{gathered} B_{n} \left( 0 \right) = - {\text{H}}\left( 0 \right)\frac{2}{L}\int_{0}^{L} {Y_{n} \left( x \right)\varphi \left( x \right){\text{d}}x} \hfill \\ \dot{B}_{n} \left( 0 \right) = - {\dot{\text{H}}}\left( 0 \right)\frac{2}{L}\int_{0}^{L} {Y_{n} \left( x \right)\varphi \left( x \right){\text{d}}x} \hfill \\ \ddot{B}_{n} \left( 0 \right) = - {\ddot{\text{H}}}\left( 0 \right)\frac{2}{L}\int_{0}^{L} {Y_{n} \left( x \right)\varphi \left( x \right){\text{d}}x} \hfill \\ \end{gathered} \right. $$

Based on Cardans formula and general solutions of nonhomogeneous linear differential equation, the solution of Eq. (36) is

40 $$ \begin{gathered} B_{n} \left( t \right) = V_{0} \left( t \right)B_{n} \left( 0 \right) - V_{1} \left( t \right)\dot{B}_{n} \left( 0 \right) + V_{2} \left( t \right)\ddot{B}_{n} \left( 0 \right) \\ + \frac{1}{a}\int\limits_{0}^{t} {f(\eta )V_{2} \left( {t - \eta } \right){\text{d}}\eta } \\ \end{gathered} $$

where *V*_0_(*t*), *V*_1_(*t*), *V*_2_(*t*) are effect functions of initial conditions on the temperature response, which has four different formulations decided by the coefficients of Eq. (36). By defining

41 $$ p = \frac{{3ac - b^{2} }}{{3a^{2} }},\;\;\;q = \frac{{2b^{3} - 9abc + 27a^{2} d}}{{27a^{3} }},\Delta = \left( \frac{q}{2} \right)^{2} + \left( \frac{p}{3} \right)^{3} , $$

the four cases are shown in the following:

*Case 1* $$\Delta < 0$$, $$\alpha + i\beta = \sqrt[3]{{ - \frac{q}{2} + i\sqrt { - \Delta } }}$$

$\alpha ,\;\beta$ are the real part and imaginary part of the complex number $\sqrt[3]{{ - \frac{q}{2} + i\sqrt { - \Delta } }}$ respectively.

42 $$ \begin{aligned} r_{1} & = 2\alpha - \frac{b}{3a} \\ r_{2} & = - \alpha - \sqrt 3 \beta - \frac{b}{3a} \\ r_{3} & = - \alpha + \sqrt 3 \beta - \frac{b}{3a} \\ \end{aligned} $$

43 $$ \begin{aligned} V_{0} \left( t \right) & = \frac{{r_{2} r_{3} e^{{r_{1} t}} }}{{\left( {r_{1} - r_{2} } \right)\left( {r_{1} - r_{3} } \right)}} + \frac{{r_{3} r_{1} e^{{r_{2} t}} }}{{\left( {r_{2} - r_{3} } \right)\left( {r_{2} - r_{1} } \right)}} + \frac{{r_{1} r_{2} e^{{r_{3} t}} }}{{\left( {r_{3} - r_{1} } \right)\left( {r_{3} - r_{2} } \right)}} \\ V_{1} \left( t \right) & = \frac{{\left( {r_{2} + r_{3} } \right)e^{{r_{1} t}} }}{{\left( {r_{1} - r_{2} } \right)\left( {r_{1} - r_{3} } \right)}} + \frac{{\left( {r_{3} + r_{1} } \right)e^{{r_{2} t}} }}{{\left( {r_{2} - r_{3} } \right)\left( {r_{2} - r_{1} } \right)}} + \frac{{\left( {r_{1} + r_{2} } \right)e^{{r_{3} t}} }}{{\left( {r_{3} - r_{1} } \right)\left( {r_{3} - r_{2} } \right)}} \\ V_{2} \left( t \right) & = \frac{{e^{{r_{1} t}} }}{{\left( {r_{1} - r_{2} } \right)\left( {r_{1} - r_{3} } \right)}} + \frac{{e^{{r_{2} t}} }}{{\left( {r_{2} - r_{3} } \right)\left( {r_{2} - r_{1} } \right)}} + \frac{{e^{{r_{3} t}} }}{{\left( {r_{3} - r_{1} } \right)\left( {r_{3} - r_{2} } \right)}} \\ \end{aligned} $$

*Case 2* $$\Delta > 0$$

44 $$ \begin{aligned} r_{1} & = \sqrt[3]{{ - \frac{q}{2} + \sqrt \Delta }} + \sqrt[3]{{ - \frac{q}{2} - \sqrt \Delta }} - \frac{b}{3a} \\ \alpha & = \frac{ - 1}{2}\left( {\sqrt[3]{{ - \frac{q}{2} + \sqrt \Delta }} + \sqrt[3]{{ - \frac{q}{2} - \sqrt \Delta }}} \right) - \frac{b}{3a} \\ \beta & = \frac{\sqrt 3 }{2}\left( {\sqrt[3]{{ - \frac{q}{2} + \sqrt \Delta }} - \sqrt[3]{{ - \frac{q}{2} - \sqrt \Delta }}} \right) \\ \end{aligned} $$

45 $$ \begin{gathered} V_{0} \left( t \right) = \frac{1}{{\left( {\alpha - r_{1} } \right)^{2} + \beta^{2} }}\left\{ {\left( {\alpha^{2} + \beta^{2} } \right)e^{{r_{1} t}} + \frac{{e^{\alpha t} }}{\beta }\left[ { - \beta \left( {2\alpha r_{1} - r_{1}^{2} } \right)\cos \beta t + r_{1} \left( {\alpha^{2} - \beta^{2} - \alpha r_{1} } \right)\sin \beta t} \right]} \right\} \\ V_{1} \left( t \right) = \frac{1}{{\left( {\alpha - r_{1} } \right)^{2} + \beta^{2} }}\left\{ {2\alpha e^{{r_{1} t}} + \frac{{e^{\alpha t} }}{\beta }\left[ { - 2\alpha \beta \cos \beta t + \left( {\alpha^{2} - \beta^{2} - r_{1}^{2} } \right)\sin \beta t} \right]} \right\} \\ V_{2} \left( t \right) = \frac{1}{{\left( {\alpha - r_{1} } \right)^{2} + \beta^{2} }}\left\{ {e^{{r_{1} t}} + \frac{{e^{\alpha t} }}{\beta }\left[ { - \beta \cos \beta t + \left( {\alpha - r_{1} } \right)\sin \beta t} \right]} \right\} \\ \end{gathered} $$

*Case 3* $$\Delta = 0$$ and $$\left( \frac{q}{2} \right)^{2} = - \left( \frac{p}{3} \right)^{3} \ne 0$$

46 $$ r_{1} = 2\sqrt[3]{{ - \frac{q}{2}}} - \frac{b}{3a},\;\;\;r_{2} = r_{3} = r = - \sqrt[3]{{ - \frac{q}{2}}} - \frac{b}{3a} $$

47 $$ \begin{gathered} V_{0} \left( t \right) = r^{2} \frac{{{\text{e}}^{{r_{1} t}} - {\text{e}}^{rt} }}{{\left( {r_{1} - r} \right)^{2} }} + {\text{e}}^{rt} + r_{1} r\frac{{t{\text{e}}^{rt} }}{{r - r_{1} }} \\ V_{1} \left( t \right) = 2r\frac{{{\text{e}}^{{r_{1} t}} - {\text{e}}^{rt} }}{{\left( {r_{1} - r} \right)^{2} }} + \left( {r + r_{1} } \right)\frac{{t{\text{e}}^{rt} }}{{r - r_{1} }} \\ V_{2} \left( t \right) = \frac{{{\text{e}}^{{r_{1} t}} - {\text{e}}^{rt} }}{{\left( {r_{1} - r} \right)^{2} }} + \frac{{t{\text{e}}^{rt} }}{{r - r_{1} }} \\ \end{gathered} $$

*Case 4* $$\Delta = 0$$ and $$p = q = 0$$

48 $$ r_{1} = r_{2} = r_{3} = r = - \frac{b}{3a} $$

49 $$ \begin{aligned} V_{0} \left( t \right) & = \left( {1 - rt + \frac{{r^{2} t^{2} }}{2}} \right)e^{rt} \\ V_{1} \left( t \right) & = \left( { - t + rt^{2} } \right)e^{rt} \\ V_{2} \left( t \right) & = \frac{{t^{2} }}{2}e^{rt} \\ \end{aligned} $$

Since the solution of $\theta_{1} \left( {x,t} \right)$ is obtained, the veritable temperature increment $\theta \left( {x,t} \right)$ is expressed as

50 $$ \theta (x,t) = \sum\limits_{n = 1}^{\infty } {Y_{n} \left( x \right)B_{n} \left( t \right)} + \left( {\frac{{e^{ - \lambda x} - e^{{\lambda \left( {x - 2L} \right)}} }}{{1 - e^{ - 2\lambda L} }}} \right)\theta_{0} \cdot {\text{H}}\left( t \right) $$

As for the case II of constant heat flux, only some modifications needs to perform to obtain the analytical solution by the method of separation of variables. With boundary condition equation (21), the solution of equation (24) can expressed as

51 $$ \varphi \left( x \right) = \frac{{e^{{ - \lambda \left( {L - x} \right)}} - e^{{\lambda \left( {L - x} \right)}} }}{{\lambda \left( {e^{ - \lambda L} + e^{\lambda L} } \right)}}\phi_{in} \left( {1 - R_{d} } \right) $$

Then the transient problem $\theta_{1} \left( {x,t} \right)$ cooperate with the following homogeneous boundary condition and nonhomogeneous initial condition equation (30).

52 $$ {\mathbf{B}}{\mathbf{.C}}{\mathbf{.}}\;\;\;\left\{ \begin{gathered} \frac{{\partial \theta_{1} }}{\partial x}(0,t) = 0 \hfill \\ \theta_{1} (L,t) = 0 \hfill \\ \end{gathered} \right. $$

The eigen function for the boundary condition is written as

53 $$ Y_{n} \left( x \right) = \cos \left( {\lambda_{n} x} \right) $$

where $\lambda_{n} = \frac{2k - 1}{2}\frac{\pi }{L},\;\;\;k = 1,2,3...$. The eigen function satisfies the following relation

54 $$ \int_{0}^{L} {Y_{n} \left( x \right)Y_{m} \left( x \right){\text{d}}x} = \left\{ \begin{gathered} \begin{array}{*{20}c} 0 & {\;n \ne m} \\ \end{array} \hfill \\ \begin{array}{*{20}c} \frac{L}{2} & {n = m} \\ \end{array} \hfill \\ \end{gathered} \right. $$

The other procedure and statement are the same as those mentioned in case I.

## Results and discussions

In this section, the analytical results would be presented and illustrated. For case I, the outer skin surface is specified to be heated to a temperature of *T*_0_ = 80 °C, and the temperature of arterial blood keeps constant as *T*_*a*_ = 37 °C. For case II, the maximum intensity of laser irradiance is considered as *Q*_0_ = 2 W/cm^2^ and the diffuse reflectance is taken as *R*_*d*_ = 0.05^28^. The thickness of the tissue is deemed as *L* = 9 mm. The thermophysical parameters of the tissue and blood are^18,29–31^: $\rho_{t} {\text{ = 1190kg/m}}^{{3}}$, $c_{t} {\text{ = 3600J/}}\left( {{\text{kg}} \cdot {\text{K}}} \right)$, $\rho_{b} {\text{ = 1060kg/m}}^{3}$,$c_{b} = 3770{\text{J/}}\left( {{\text{kg}} \cdot {\text{K}}} \right)$,$\omega_{b} = 1.87 \times 10^{ - 3} {\text{s}}^{{ - 1}}$,$k = 0.235{\text{W/}}\left( {{\text{m}} \cdot {\text{K}}} \right)$,$k^{ * } = 0.1{\text{W/}}\left( {{\text{m}} \cdot {\text{K}} \cdot {\text{s}}} \right)$. The three phase lags $\tau_{q}$, $\tau_{T}$ and $\tau_{v}$ may be assumed as different values in the following discussions, and their values will be given together with the discussions. The rate of thermal conductivity hasn’t been studied in bio-transfer of biological tissue before, so the constant value of the constant rate of conductivity is assumed based on request of solution^18^ and relative value to thermal conductivity^23,25^.

### Convergence of series solutions

**Figure 1:**
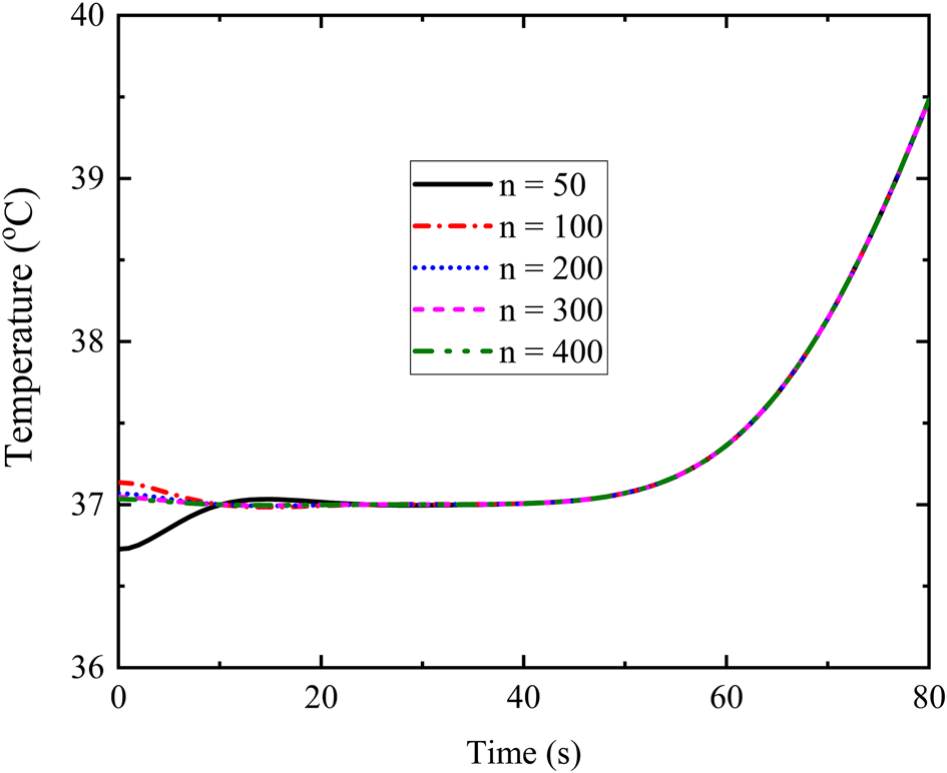
Time-history of temperature for different number of series solutions at *x* = 0.5*L*.

Figure 1 depicts the time-history of temperature with different number of terms in series solutions in TPL model. The phase lags are *τ*_*q*_ = 16 s, *τ*_*T*_ = 6 s^32^ and *τ*_*v*_ = 2 s^18^, which satisfy the inequality 0 ≤ *τ*_*v*_ < *τ*_*T*_ < *τ*_*q*_^14^. It is necessary to mention that *τ*_*v*_ is assumed as 2 s based on the stable request of solution^18^ and relative size of numerical value between the phase lags of heat flux, temperature gradient and thermal displacement gradient^14,22,24^. It is shown in the figure that the results converge quickly as the number increases. The curves for the cases of n = 300 and n = 400 are almost coincided together, so 300 terms in series solution could be adopted in the present calculation and analysis.

### Validation of the solving method

**Figure 2:**
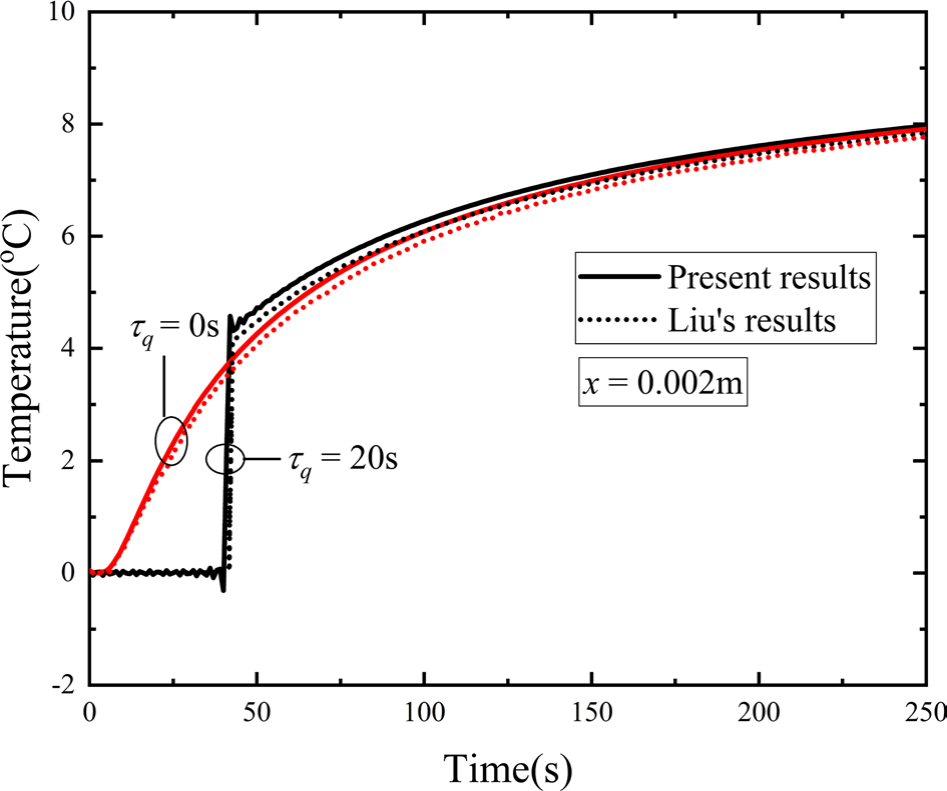
Comparison of the present model and Liu’s results.

To check the validation of the solution procedure and method used in this paper, the same problem in Liu’s work^11^ is calculated with the same parameters being used. According to reference^11^, the following parameters are applied: *ρ*_*t*_ = 1000 kg/m^3^, *c*_*t*_ = 4200 J/(kg·K), *ρ*_*b*_ = 1000 kg/m^3^, *c*_*b*_ = 4200 J/(kg·K), *k* = 0.2 W/(m·K), *ω*_*b*_ = 0.5 × 10^-3^ s^-1^, and *L* = 0.0012 m. These parameters are just used in this subsection. The comparison between the present results and Liu’s results is depicted in Fig. 2.

In Liu’s work^11^ the Pennes model and C-V model are adopted. In this present model, with assumption of *τ*_*v*_ = 0 s and *τ*_*T*_ = 0 s, the model can reduce to C-V bio-heat transfer model. Furtherly, it reduces to Pennes model when *τ*_*q*_ = 0 s. The time history of temperature at the location of *x* = 0.002 m for the Pennes model and C-V model of present model and Liu’s model are shown in Fig. 2. Temperature in the skin increases immediately when the outer surface is heated. The C-V model is adopted with phase lag of heat flux taken as *τ*_*q*_ = 20 s. It is shown that the temperature doesn’t change at once, while it jumps to a high value at the time of *t* = 41 s. The thermal wave speed in C-V model is given by $V_{C - V} = \sqrt {{k \mathord{\left/ {\vphantom {k {\tau_{q} \rho_{t} c_{t} }}} \right. \kern-\nulldelimiterspace} {\tau_{q} \rho_{t} c_{t} }}}$, which gives the value of $4.88 \times 10^{ - 5} {{\text{m}} \mathord{\left/ {\vphantom {{\text{m}} {\text{s}}}} \right. \kern-\nulldelimiterspace} {\text{s}}}$ under present configuration. At the time of *t* = 41 s, the thermal wave arrives at the location of *x* = *V·t* = 0.002 m, which is just the location under study in Fig. 2. Good agreement is obtained between this work and Liu’s results in Fig. 2. Hence the method used in present study is efficient and will be adopted in the following calculations.

### Parametric study of influences on temperature response in TPL model for the case of constant surface temperature

#### Comparison between TPL model and former models

**Figure 3:**
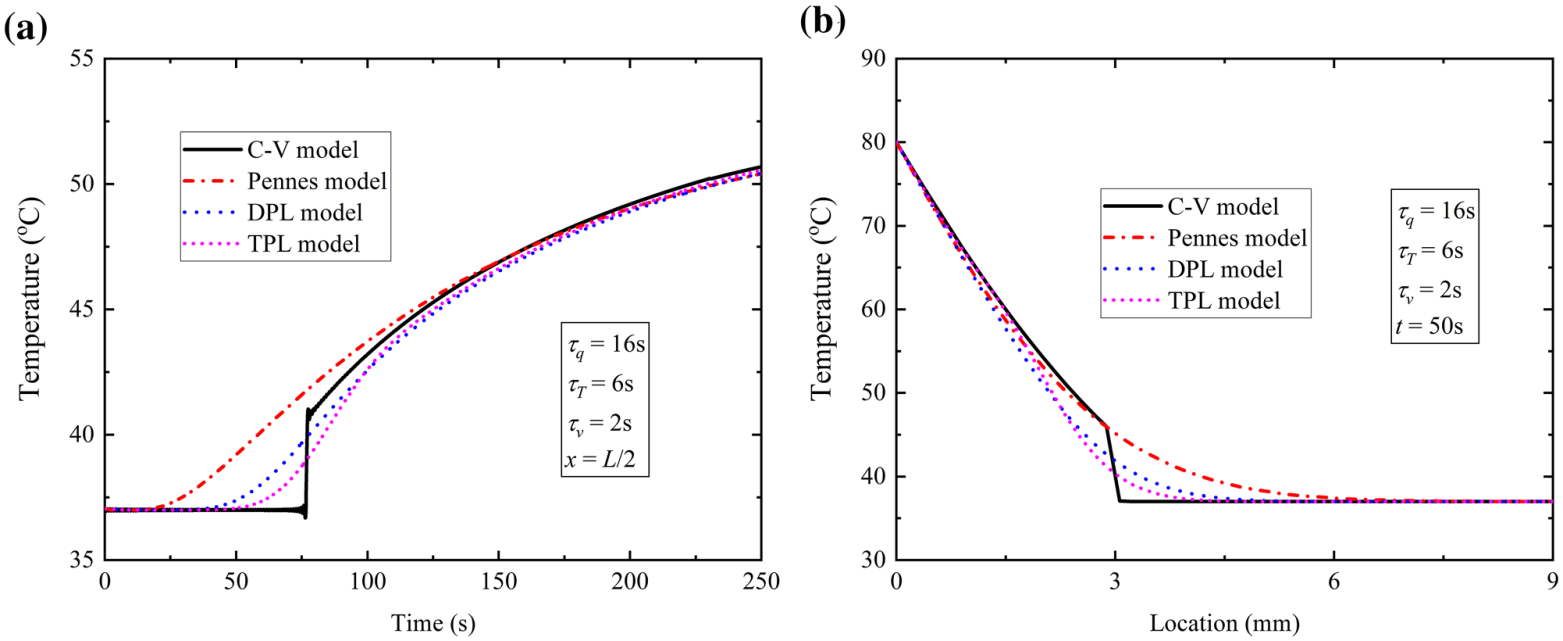
Results of different heat conduction models for the case of constant surface temperature. (**a**) time-history of temperature at location *x* = *L*/2; (**b**) temperature distribution at time *t* = 50 s.

Figure 3 shows the comparison of temperature response of the TPL model, Pennes model, C-V model and DPL model. Figure 3a shows the time-history of temperature at the location *x* = 0.5*L* for the bio-heat models under discussion. It is found that the thermal propagation speed is the largest in Pennes model and C-V model predicts the lowest one. It is apparent that the curve in TPL model is between the curves of C-V model and DPL model, which means that the TPL model can show both the diffusion and wave characteristics of bio-heat transfer. The thermal propagation speed in TPL model is smaller than that in DPL model so that the thermal response in TPL model occurs later than that in DPL model. Figure 3b depicts the temperature distribution for the above models at the time *t* = 50 s. It can be seen that there is a drop of temperature in C-V model, which shows the location where the thermal wave arrives. Before this location, the temperature in C-V model is larger than those in the other three models, while the temperature predicted in Pennes model is the largest after this location. These could be reasonable because in C-V model the lowest thermal propagation speed would lead to more heat concentrated in the affected area by surface heating and on the contrary, the more thermal energy would transfer to the unaffected zone quickly due to the infinite wave propagation speed in Pennes model.

#### *Effect of τ*_*v*_* on temperature response*

**Figure 4:**
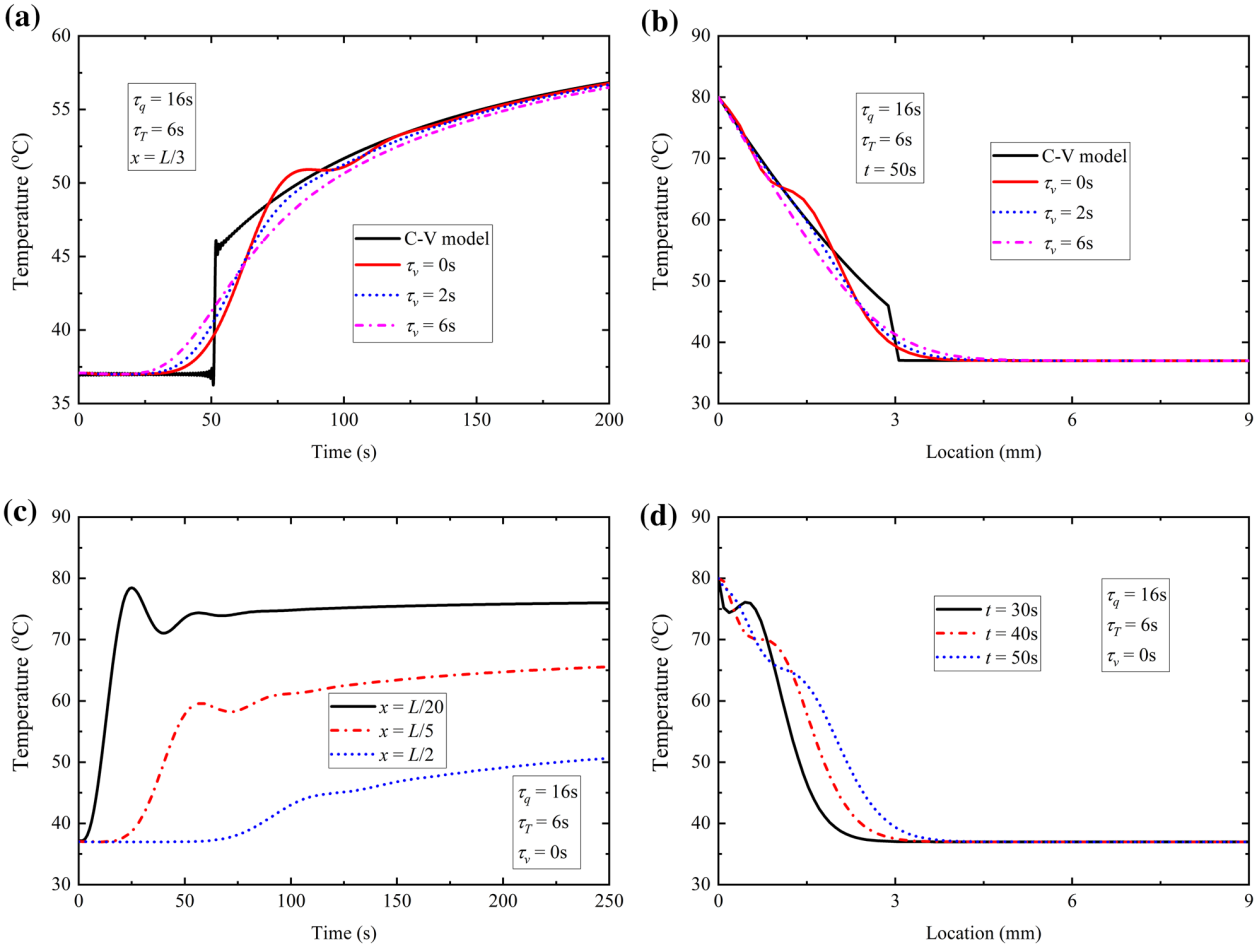
Effect of *τ*_*v*_ with constant surface temperature. (a) time-history of temperature at location *x* = *L*/3; (b) temperature distribution at time *t* = 50 s; (c) time-history of temperature at different location with *τ*_*v*_ = 0 s; (d) temperature distribution at different time with *τ*_*v*_ = 0 s.

Due to the uninvestigated problem in biological tissue and lack of specific value for the phase lag of thermal displacement gradient, the influence of the phase-lag of thermal displacement gradient *τ*_*v*_ needs to be investigated and the results are shown in Fig. 4. In present paper, all the values of phase lag times attempt to be determined by the stable request of temperature solution^18^, the fundamental inequality 0 ≤ *τ*_*v*_ < *τ*_*T*_ < *τ*_*q*_^14,23^.

Figure 4a shows variation of temperature with different phase-lag *τ*_*v*_ for TPL model at the location *x* = *L*/3. For comparison, the result of C-V model is also shown in the figure. Figure 4b depicts the temperature distribution at the time *t* = 50 s for different phase-lags of thermal displacement gradient. It can be found that the temperature response behavior would get closer to the C-V model of wave characteristic when the phase-lag of thermal displacement gradient *τ*_*v*_ is getting smaller. It is apparent that the decreasing of *τ*_*v*_ requires more time to motivate thermal response at the same location from Fig. 4a and the larger *τ*_*v*_ leads to the longer thermal propagation distance at the same time from Fig. 4b. That indicates the increasing of *τ*_*v*_ would increase the thermal propagation speed and accelerate thermal propagation process so that there would be more thermal energy transfer to the unaffected area from the affected zone. Therefore it can be seen from Fig. 4b that the temperature with large *τ*_*v*_ is lower than that with small *τ*_*v*_ at the location near to the surface while temperature is lower with small *τ*_*v*_ away from the surface.

It is worthwhile noticing that small oscillation occurs in temperature response when *τ*_*v*_ is zero, which hasn’t been shown in former bio-heat transfer model before. This phenomenon is more obvious at the location nearer to the heating surface in Fig. 4c,d which results from the thermal propagation wave by instantaneous heating. The wave characteristic in space is similar to the vibration phenomenon of a string motivated by constant transverse displacement at left end abruptly in Fig. 4d. Therefore the wave characteristic would be more obvious for heating way of instant temperature rise when phase lag of thermal displacement gradient is taken as zero (*τ*_*v*_ = 0 s). This indicates the phase lag of thermal displacement gradient will be small if the wave characteristic of bio-heat transfer in some biological tissue and the phase lag ${\tau }_{v}$ is significant in bring about the oscillation characteristic.

#### Effects of τ_q_ and τ_T_ on temperature response

**Figure 5:**
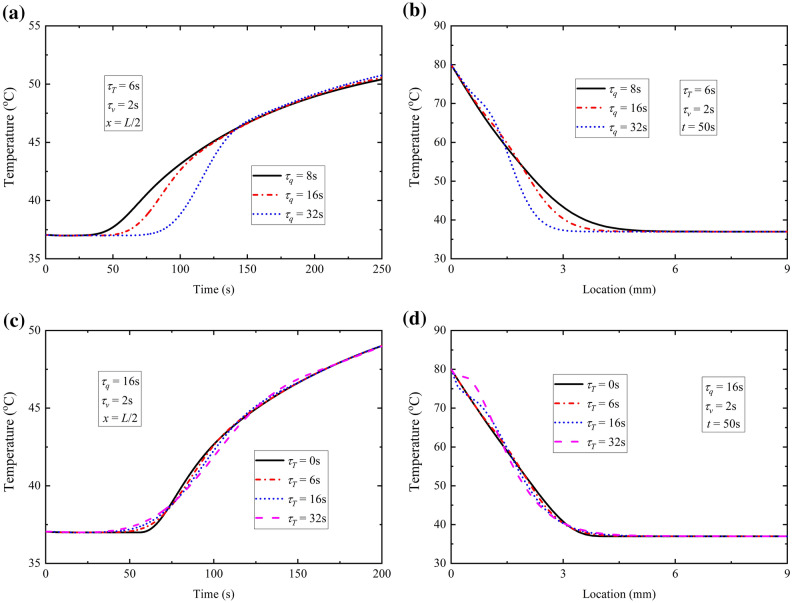
Effects of *τ*_*q*_ and *τ*_*T*_ with constant surface temperature. (**a**) Time-history of temperature at location *x* = *L*/2 with different *τ*_*q*_; (**b**) temperature distribution at time *t* = 50 s with different *τ*_*q*_; (**c**) time-history of temperature at location *x* = *L*/2 with different *τ*_*T*_ ; (**d**) temperature distribution at time *t* = 50 s with different *τ*_*T*_.

The effect of τ_q_ on temperature response is illustrated in Fig. 5a,b. As mentioned in C-V model, τ_q_ indicates a lag phenomenon of heat flux which means thermal energy would not transfer instantly. The larger τ_q_ is, the more obvious the lagging characteristic of thermal behavior, which results in higher temperature in affected area (Fig. 5b) and steeper rise of temperature (Fig. 5a). Comparing with τ_q_, the influence of τ_T_ is smaller on lag characteristic and thermal propagation speed, which is shown in Fig. 5c,d.

#### *Effect of ω*_*b*_* on thermal response*

**Figure 6:**
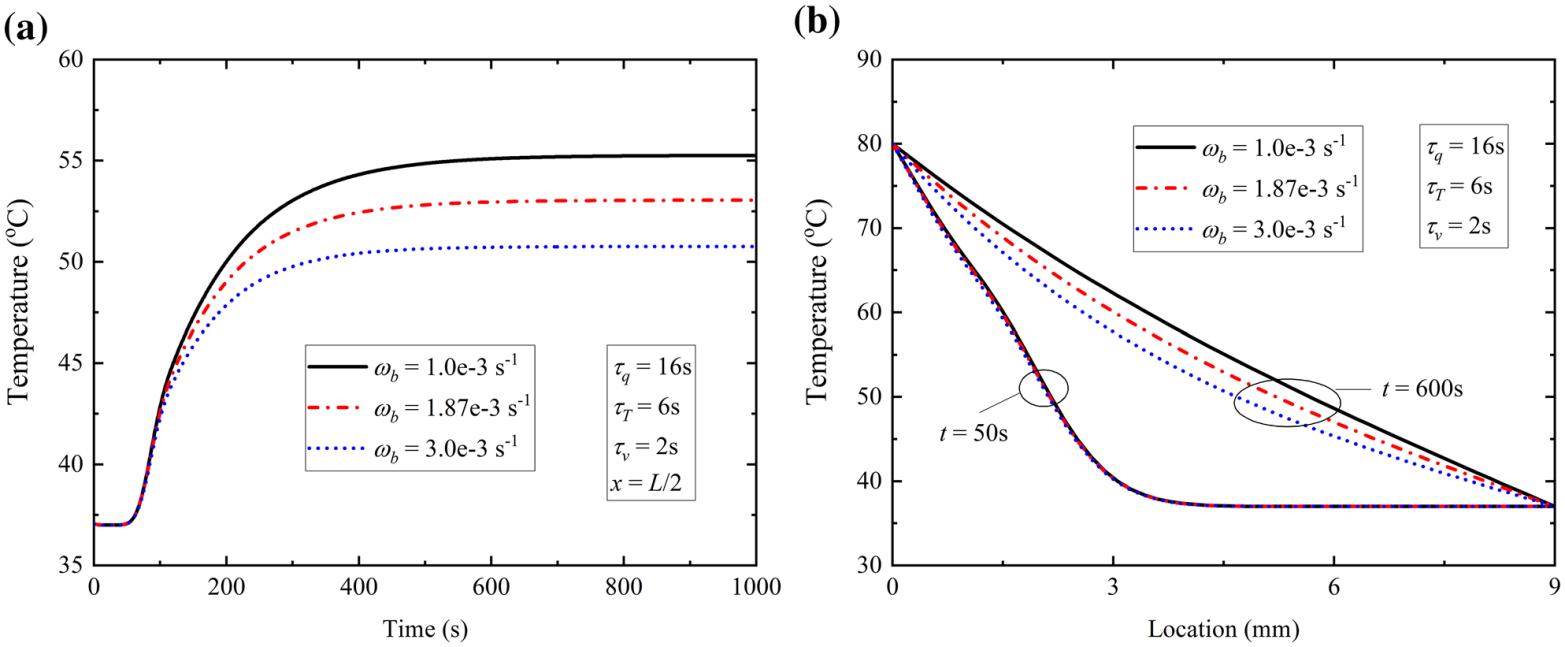
Effect of the perfusion rate of blood on (**a**) time-history of temperature at location *x* = *L*/2; (**b**) temperature distributions at time *t* = 50 s and *t* = 600 s.

Finally, the influence of the perfusion rate of blood *ω*_*b*_ is shown in Fig. 6. The development of temperature in TPL model for different perfusion rates of blood *ω*_*b*_ at location *x* = *L*/2 are shown in Fig. 6a and the corresponding temperature distribution at time *t* = 50 s and *t* = 600 s are shown in Fig. 6b which represents the transient and steady states in heat transfer. The most obvious characteristic of the effect of *ω*_*b*_ on temperature is that the increasing of *ω*_*b*_ could decrease temperature of steady state after time *t* = 600 s from Fig. Figure 6a and has no obvious effect on Non-Fourier effect at time *t* = 50 s from Fig. 6b. It could be reasonable and acceptable that heat transfer quantity increases with the increasing of perfusion rate of blood because the flowing blood would take more thermal energy away from subjected skin tissue which leads to the lower steady-state temperature in local tissue.

### Parametric study of influences on thermal behavior in TPL model for case of constant surface heat flux

Due to limitation of the paper length, only the influences of τ_v_ and ω_b_ on temperature response are discussed for this case.

**Figure 7:**
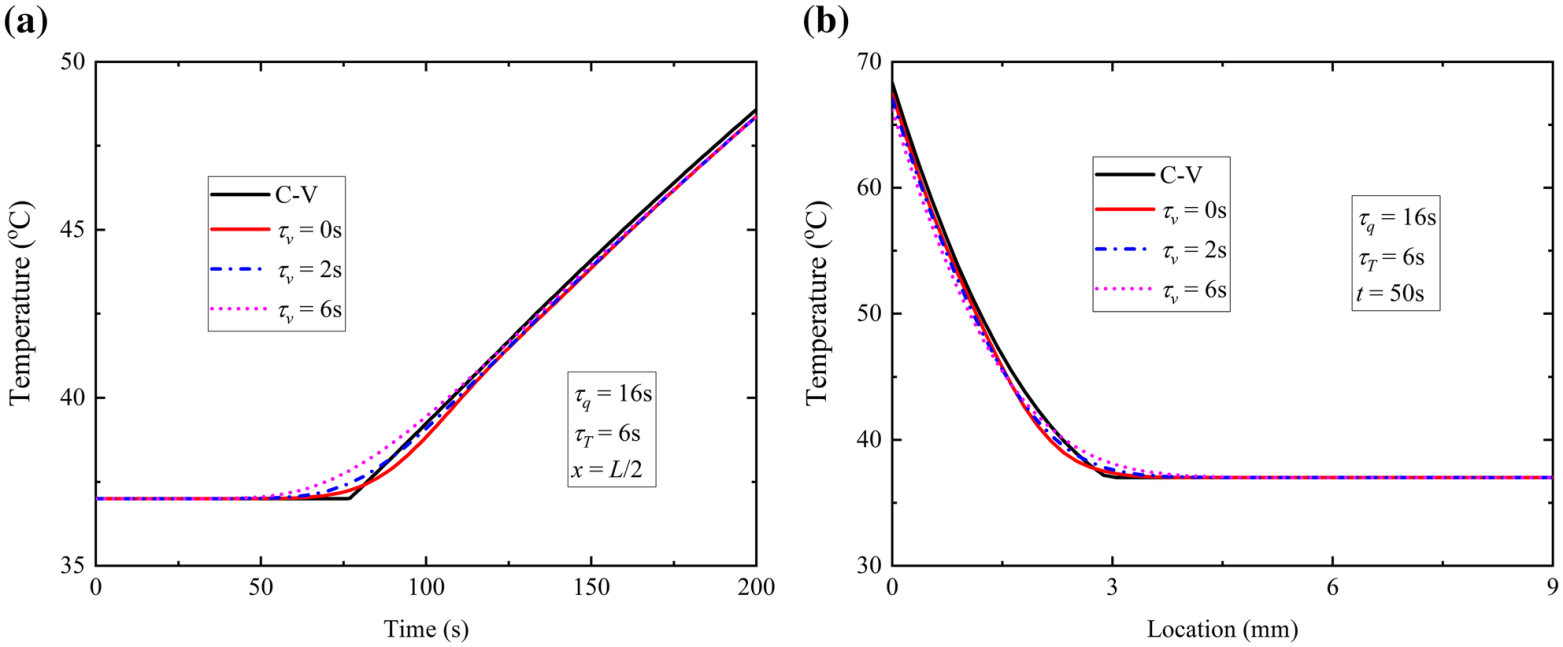
Temperature response for different *τ*_*v*_ with constant surface heat flux.

Figure 7a depicts the variation of temperature at *x* = *L*/2 with different phase lag times of thermal displacement gradient. Reduction of $\tau_{v}$ makes the variation of temperature closer to C-V model but in a smoother way, which indicates the increasing of *τ*_*v*_ could enhance the speed of thermal propagation a little. This could be concluded in Fig. 7b which plots the temperature distribution at time *t* = 50 s. However the discrepancy with Fig. 4 is the oscillation phenomenon doesn’t arise under the present configuration, which is attributed to the difference of boundary conditions. A specific temperature is applied on the surface of skin tissue in Fig. 4, while a specific heat flux is loaded on the surface in Fig. 7. One case is field variable increment, while the other is gradient rise of the corresponding field variable which leads to gentle increment of the field variable rather than abrupt increment or oscillation increment.

**Figure 8:**
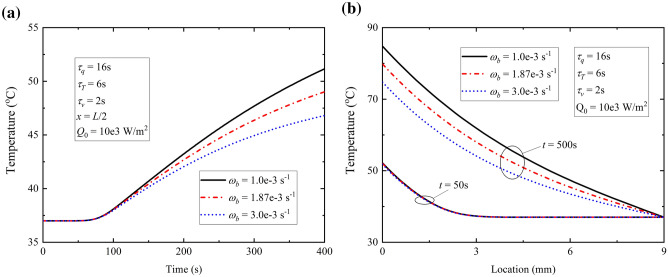
Temperature response for different ω_b_ with constant surface heat flux.

The effect of ω_b_ on temperature response is shown in Fig. 8. Blood perfusion in energy conservation equation means the heat transfer to blood from affected skin tissue. Hence enhancement of ω_b_ represents more blood flowing and participating heat convection, which leads to lower temperature in the same location at the same time, as is shown in Fig. 8a. In Fig. 8b no difference between different blood perfusion rate at time *t* = 50 s is attributed to fact that the blood perfusion primarily affects the stable solution of temperature according to equation (25) and hardly shows discrepancy in short time transient process.

## Conclusions

The thermal response behavior in a one-dimensional skin tissue model which is subjected to surface heating and heat source of laser irradiance is investigated based on the TPL bio-heat conduction model. By combining the TPL heat conduction equation and the modified energy conservation equation, a new temperature governing equation has been obtained in the heat transfer process of skin tissue. The influences of the phase-lags of heat flux, temperature gradient and thermal displacement gradient and the rate of perfusion of arterial blood on temperature responses are investigated emphatically. The comparison between TPL, DPL and C-V and Pennes models has been made to find more attractive phenomenon about TPL bio-heat conduction theory. The following conclusions can be drawn from the results and analysis:

- The new bio-heat conduction model with three phase-lags predicts both diffusion and wave characteristics of bio-heat transfer. Therefore the behavior of temperature obtained from TPL model is between that of the C-V model and DPL model.
- Increasing of the phase-lag of heat flux determines the lagging characteristic of thermal behavior and the phase lag of temperature gradient would gentle the process and temperature rise.
- The phase lag of thermal displacement gradient *τ*_*v*_ is helpful to decrease local tissue temperature. And temperature increment oscillates for a while with constant temperature of surface boundary condition when the phase lag of thermal displacement gradient is assumed as zero (*τ*_*v*_ = 0).
- The increase of the rate of perfusion of arterial blood *ω*_*b*_ leads to more heat taken away from local affected tissues resulting in lower temperature of steady state.

## Supplementary information

Supplementary Information.

## Data Availability

The raw/processed data required to reproduce these findings cannot be shared at this time due to technical or time limitations.
