# Supplementary material for: Bio-heat response of skin tissue based on three-phase-lag model
Source: Sci Rep. 2020 Oct 2;10:16421. doi: 10.1038/s41598-020-73590-3 (PMC7532225; doi:10.1038/s41598-020-73590-3)
Supplement: Supplementary file 1 — Supplementary Information. [file 41598_2020_73590_MOESM1_ESM.docx]

**Bio-heat response of skin tissue based on three-phase-lag model**

Qiao Zhang, Yuxin Sun^*^, Jialing Yang

Institute of Solid Mechanics, School of Aeronautic Science and Engineering, Beihang University, Beijing 100191, P.R. China

^*^ Corresponding author. E-mail address: yxsun@buaa.edu.cn.

# Appendix Modification of energy conservation equation

The classical energy conservation equation is proposed by Pennes as

$\rho_{t}c_{t}\frac{\partial T}{\partial t}=-\nabla\cdot\vec{q}+\omega_{b}\rho_{b}c_{b}\left(T_{a}-T\right)+Q_{m}+Q_{laser}$ (A.1)

By Taylor’s expansion up to the first term in phase-lag of heat flux or temperature gradient, the heat flux constitutive equation can be expressed as

Pennes model:

$\vec{q}=-k\nabla T$ (A.2)

C-V model:

$\left(1+\tau_{q}\frac{\partial}{\partial t}\right)\vec{q}=-k\nabla T$ (A.3)

DPL model:

$\left(1+\tau_{q}\frac{\partial}{\partial t}\right)\vec{q}=-k\left(1+\tau_{T}\frac{\partial}{\partial t}\right)\nabla T$ (A.4)

TPL model:

$\left(1+\tau_{q}\frac{\partial}{\partial t}\right)\vec{q}=-k\nabla\left[\left(1+\tau_{T}\frac{\partial}{\partial t}\right)T+\frac{k^{*}}{k}\left(1+\tau_{v}\frac{\partial}{\partial t}\right)v\right]$ (A.5)

or

$\left(\frac{\partial}{\partial t}+\tau_{q}\frac{\partial^{2}}{\partial t^{2}}\right)\vec{q}=-\left[k\tau_{T}\frac{\partial^{2}}{\partial t^{2}}+\left(k+k^{*}\tau_{v}\right)\frac{\partial}{\partial t}+k^{*}\right]\nabla T$ (A.6)

By combining the heat flux constitutive equations and the energy equation (A.1), the following governing equations related to temperature increment without volume heat source could be obtained as

Pennes model:

$\rho_{t}c_{t}\frac{\partial\theta}{\partial t}+\omega_{b}\rho_{b}c_{b}\theta=k\nabla^{2}\theta$ (A.7)

C-V model:

$\tau_{q}\rho_{t}c_{t}\frac{\partial^{2}\theta}{\partial t^{2}}+\left(\rho_{t}c_{t}+\tau_{q}\omega_{b}\rho_{b}c_{b}\right)\frac{\partial\theta}{\partial t}+\omega_{b}\rho_{b}c_{b}\theta=k\nabla^{2}\theta$ (A.8)

DPL model:

$\tau_{q}\rho_{t}c_{t}\frac{\partial^{2}\theta}{\partial t^{2}}+\left(\rho_{t}c_{t}+\tau_{q}\omega_{b}\rho_{b}c_{b}\right)\frac{\partial\theta}{\partial t}+\omega_{b}\rho_{b}c_{b}\theta=k\left(1+\tau_{T}\frac{\partial}{\partial t}\right)\nabla^{2}\theta$ (A.9)

TPL model:

$\begin{matrix} \tau_{q}\rho_{t}c_{t}\frac{\partial^{3}\theta}{\partial t^{3}}+\left(\rho_{t}c_{t}+\tau_{q}\omega_{b}\rho_{b}c_{b}\right)\frac{\partial^{2}\theta}{\partial t^{2}}+\omega_{b}\rho_{b}c_{b}\frac{\partial\theta}{\partial t}= \\ \left[k\tau_{T}\frac{\partial^{2}}{\partial t^{2}}+\left(k+k^{*}\tau_{v}\right)\frac{\partial}{\partial t}+k^{*}\right]\nabla^{2}\theta\end{matrix}$ (A.10)

If the outer surface is heated to a given temperature increment $\theta_{0}$, the temperature increment solution of steady state needs to satisfy the following equations for the four models, respectively:

Pennes, C-V and DPL model

$\omega_{b}\rho_{b}c_{b}\theta=k\nabla^{2}\theta$ (A.11)

TPL model

$\nabla^{2}\theta=0$ (A.12)

Obviously, the solution in TPL model is different from that of the Pennes model which is efficient and correct in analyzing heat conduction of steady state for long heating duration. So some modifications to the TPL model are necessary to obtain the correct solution of steady state.

The modification is based on the heat flux constitutive equation (A.5) in which the temperature and the thermal displacement could be regarded as an integral variable like DPL model (A.4). Hence by substituting the integral variable$\left(T+\left(k^{*}/k\right)v\right)$instead of temperature variable *T* in equation (A.1), the following energy equation could be obtained as

$\rho_{t}c_{t}\frac{\partial}{\partial t}\left(T+\frac{k^{*}}{k}v\right)=-\nabla\cdot\vec{q}+\omega_{b}\rho_{b}c_{b}\left[T_{a}-T+\frac{k^{*}}{k}\left(v_{a}-v\right)\right]+Q_{m}+Q_{laser}$ (A.13)

By combining with the heat flux constitutive equation, the modified temperature increment governing equation could be expressed as

$\begin{matrix} \tau_{q}\rho_{t}c_{t}\frac{\partial^{3}\theta}{\partial t^{3}}+\left[\rho_{t}c_{t}\left(1+\tau_{q}\frac{k^{*}}{k}\right)+\tau_{q}\omega_{b}\rho_{b}c_{b}\right]\frac{\partial^{2}\theta}{\partial t^{2}}+\left[\rho_{t}c_{t}\frac{k^{*}}{k}+\omega_{b}\rho_{b}c_{b}\left(1+\tau_{q}\frac{k^{*}}{k}\right)\right]\frac{\partial\theta}{\partial t} \\ +\omega_{b}\rho_{b}c_{b}\frac{k^{*}}{k}\theta=\left[k\tau_{T}\frac{\partial^{2}}{\partial t^{2}}+\left(k+k^{*}\tau_{v}\right)\frac{\partial}{\partial t}+k^{*}\right]\nabla^{2}\theta\end{matrix}$(A.14)

It would reduce to steady state issue without considering derivation terms with respect to time：

$\omega_{b}\rho_{b}c_{b}\frac{k^{*}}{k}\theta=k^{*}\nabla^{2}\theta$ or $\frac{\omega_{b}\rho_{b}c_{b}}{k}\theta=\nabla^{2}\theta$ (A.15)

which predicts the same temperature distribution of steady state as Pennes model does.
